# The global temperature-related mortality impact of earlier decarbonization for the Australian health sector and economy: A modelling study

**DOI:** 10.1371/journal.pone.0271550

**Published:** 2022-08-03

**Authors:** Siddhanth Sharma, R. Daniel Bressler, Anand Bhopal, Ole F. Norheim

**Affiliations:** 1 Bergen Centre for Ethics and Priority Setting, University of Bergen, Bergen, Norway; 2 School of International and Public Affairs, Columbia University, New York, NY, United States of America; Xiamen University, CHINA

## Abstract

**Background:**

Sustained elevated concentration of GHGs is predicted to increase global mortality. With the Australian health sector responsible for 7% of the nation’s GHG emissions, the benefits and costs of various decarbonisation trajectories are currently being investigated. To assist with this effort, we model the impact earlier decarbonisation has on temperature-related mortality.

**Design:**

We used DICE-EMR, an Integrated Assessment Model with an endogenous mortality response, to simulate Australian GHG trajectories and estimate the temperature-related mortality impact of early decarbonisation. We modelled a linear decline of the Australian health sector’s and economy’s GHG annual emissions to net-zero targets of 2040 and 2050.

**Main outcome measure:**

Deaths averted and monetary-equivalent welfare gain.

**Results:**

Decarbonisation of the Australian health sector by 2050 and 2040 is projected to avert an estimated 69,000 and 77,000 global temperature-related deaths respectively in a Baseline global emissions scenario. Australian economy decarbonisation by 2050 and 2040 is projected to avert an estimated 988,000 and 1,101,000 global deaths respectively. Assuming a low discount rate and high global emissions trajectory, we estimate a monetary equivalent welfare gain of $151 billion if the Australian health sector decarbonises by 2040, only accounting for the benefits in reducing temperature-related mortality.

**Conclusions:**

Earlier decarbonisation has a significant impact on temperature-related mortality. Many uncertainties exist and health impacts other than temperature-related mortality are not captured by this analysis. Nevertheless, such models can help communicate the health risk of climate change and improve climate policy decision making.

## Introduction

Climate change is projected to have a significant effect on health. A rise in average surface temperature in response to a sustained elevated concentration of greenhouse gases (GHGs) is predicted to increase global mortality related to diarrhoeal disease, malnutrition, coastal flooding, heat illness and cardiovascular disease [1–3]. The risk to health, alongside the risk to the economy and the environment, provides society a strong impetus to achieve CO_2_ and GHG emission neutrality (i.e. net zero emissions). All sectors of the economy must change their practices to achieve this common goal, including the health sector.

Lenzen and colleagues estimated the global health sector is responsible for 4.4% of global GHG emissions [4]. The health sector in Australia comprises a higher proportion of emissions than average, estimated to contribute 7% of national GHG emissions [5]. In recognition of the health and wellbeing risk posed by climate change, multiple Australian states and territories have committed to decarbonising by 2050 [6]. Western Australia, for example, led the first statutory enquiry into the relationship between health and climate, and committed to developing a Sustainable Health Unit to assist in their bid to achieve carbon neutrality by 2050 [7].

Yet, some experts claim this is not enough. England’s National Health Service (NHS) is aiming to achieve “net zero” emissions by 2040 for the care it provides [8]. Leaders of the Australian medical community have urged policymakers to follow the NHS’ lead and target decarbonisation by 2040 [9]. A 2040 target increases the likelihood Australia meets their economy wide national targets and fulfils its responsibilities under the Paris Agreement. What is not clear, however, is how much the health sector should be willing to spend to reach the 2040 target, particularly when there is no price on carbon in Australia. Over the next two decades affordable technologies may emerge enabling a transition that does not demand significant investment—but this is not guaranteed.

Recently, in a study entitled *The Mortality Cost of Carbon* published in *Nature Communications*, Bressler created a coupled climate-economy-demographics Integrated Assessment Model (IAM) that includes a climate-mortality damage function [10]. This model extends the Dynamic Integrated Climate-Economy Model 2016 (DICE-2016), an IAM created by Nobel Prize-winning economist William Nordhaus [11]. IAMs integrate a model of the economy with a model of the climate. They include a damage function that projects how damaging different warming scenarios are on society. They are used widely to estimate the social cost of carbon (the economic cost of an additional ton of CO_2_-equivalent emission) and identify mitigation trajectories that maximise social welfare [11].

Bressler found climate-mortality damages were largely left out of DICE-2016 [10]. He therefore extended the model to include a temperature-related mortality damage function in addition to the original DICE-2016 damage function (Dynamic Integrated Climate-Economy Model with an Endogenous Mortality Response [DICE-EMR]). The mortality damage function represents the percentage increase in the global mortality rate as a function of the increase in global average temperatures, and was estimated by fitting a curve through projections made by scientific studies that were chosen from a systematic research synthesis. DICE-EMR can be used to determine the effect of GHG emissions decisions on future temperature-related excess deaths. We use DICE-EMR to assess how many additional deaths will result from GHG emissions if the Australian health sector and the whole economy follow [1] ‘Business as usual (BAU),’ or decarbonise by [2] 2050 and [3] 2040.

## Methods

### Overview

Our analysis uses DICE-EMR to simulate the following steps:

1. The projected cumulative global CO_2_ emissions emitted over the century, assuming the current emissions trend continues.
2. The effect CO_2_ emission concentration has on average surface temperature.
3. The effect a rise in average surface temperature has on the mortality rate and excess deaths.
4. The isolated effect the Australian healthcare system’s and the whole Australian economy’s CO_2_ -equivalent emissions have on the global mortality rate and excess deaths.

### Projected GHG emissions and climate sensitivity

The projected CO_2_ emissions and the climate model are taken directly from DICE-2016 [11]. Further details can be found in S1 Appendix. In brief, the projections estimate cumulative emissions based on GDP projections, the projected carbon intensity of economic output, the CO_2_ emissions of land, and the amount of CO_2_ abatement. In DICE-2016, GDP per capita is projected to increase at 2.1% per year from 2015 to 2050, and 1.9% from 2050 to 2100. The DICE Baseline emissions scenario assumes the carbon intensity of economic output decreases by 1.5% per year. The effect CO_2_ has on surface temperature is captured by a three-reservoir climate model and aligns closely with the IPCC estimates.

### Temperature effect on mortality

The following equation from DICE-EMR represents the effect of the global mean surface temperature on mortality:

$$ED_{\;\text{GC}}=\sum_{t=2020}^{t=2100}L\left(t\right)d\left(t\right)\delta T\left(t\right)-L\left(t\right)d\left(t\right)$$

Where *ED*_GC_ is the number of cumulative global excess deaths between 2020 and 2100. The number of excess deaths in each time period is derived by calculating the effect a rise in average surface temperature has on the mortality rate. *L*(*t*) is the population in period *t*, *d*(*t*) is the mortality rate and *δT*(*t*) is the mortality damage function, which represents the % increase in mortality as a function of the rise in global mean surface temperature. Future population growth, which is a function of projected fertility and mortality rates, is extracted from the 2019 United Nations World Population Prospects [12].

Bressler conducted a systematic review of 100 studies in the climate-mortality literature to find studies that were of sufficient quality and relevance to be used to estimate the mortality damages to increases in global average temperatures [10]. Ultimately, three studies were used [1–3]. Two of the three studies [1, 2] used to estimate the mortality damage function made their projections net of the projected effect of defensive adaptation, since individuals and societies are expected to make choices to reduce their vulnerability to the mortality effects of future climate change, such as by purchasing air conditioning. Although the third study [3] did not incorporate the effect of adaptation, its mortality projections were lower in large part because it did not include data from regions most vulnerable to heat related mortality in South Asia, the Middle East and Africa.

Bressler then conducted a curve-fitting exercise to estimate the mortality damage function from the projections made in these studies [10]. The mortality damage function was robust across a wide variety of functional forms, and a quadratic functional form was used in the main specification:

$$\delta T\left(t\right)=\beta_{1}T\left(t\right)+\beta_{2}T\left(t^{2}\right)$$

where *T*(*t*) is the increase in global average atmospheric temperatures above pre industrial levels and *β*_i_{*β*_1_, *β*_2_} are the estimated coefficients. For our mortality damage function, we use Bressler’s central estimate [10]. Further details of his systematic research synthesis of the climate mortality literature and curve-fitting exercise can be found in S1 Appendix.

### Contribution of the Australian health system and economy to temperature induced mortality

To estimate the contribution of the Australian health system to temperature induced mortality under different decarbonising trajectories (BAU, 2050 and 2040), the annual health system’s CO_2_ equivalent emissions (*E*_AHS_ (*t*)) are taken as a proportion of global annual CO_2_ emissions (*E*_G_ (*t*)) in Baseline DICE-EMR:

$${\text{ED}}_{\text{AHS}}=\sum_{t=2020}^{t=2100}\;ED_{\text{G}}\;\left(t\right)*\frac{E_{\text{AHS}}\;\left(t\right)}{{E\;}_{\text{G}}\;\left(t\right)}$$

**Fig 1:**
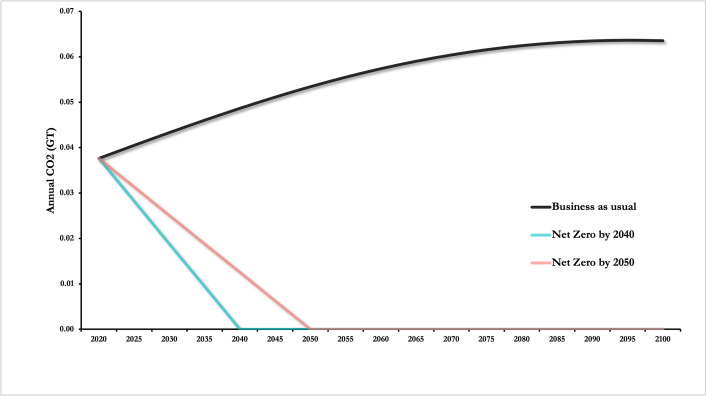
Australian health sector annual greenhouse gas emissions for hypothetical trajectories used in the model DICE-EMR to estimate the number of global deaths averted.

The Australian health system emissions for 2020 were assumed to contribute 7% of Australia’s total CO_2_ -equivalent emissions as estimated by Malik et al. [5], and decline linearly to its final target (for BAU, it was assumed to maintain a constant 7% contribution of Australia’s total CO_2_ -equivalent emissions, see Fig 1). The same equation was used to estimate the number of excess deaths averted if the economy decarbonised by 2050 and 2040. Estimates for Australia’s national emissions were taken from the National Greenhouse Gas Inventory for 2018 [13].

We also simulate the expected harm under a global low-emissions scenario, in which countries take significant action to reduce their carbon intensity, more so than suggested by recent trends. To model this we use the DICE-EMR Optimal trajectory, [10] reflecting full global decarbonisation by 2050.

### Monetising deaths averted and discounting

Finally, we monetise the present value of averted future deaths:

$${\text{NPV}}_{\text{ED}}=\sum_{t=2020}^{t=2100}\text{ED}\left(\text{t}\right){*\text{VSL}\;}_{\text{US}\;\text{2020}}\;*\left(\;\frac{Global\;GNI\;pc\;\left(t\right)}{US\;GNI\;pc\;2020}\right){}^{Income\;elasticity}\;{\left(\;\frac{1}{1+r}\right)}^{t-2020}$$

The number of excess deaths in each time period (ED(t))is multiplied by a global average value of statistical life (VSL). We derive a global VSL by using a benefits transfer methodology as discussed in Robinson, Hammitt & O’Keeffe [14]–it is a product of the US VSL (11.5 million USD) and the ratio of the median global GNI per capita and the US GNI per capita in 2020 in each time period. To convert into net present value (NPV_ED_), we discount [$(\frac{1}{1+r}){}^{t-2020}$ where *r* is the discount rate and *t* is the year excess deaths take place] by 3.5% and 5%, as recommended by the Australian Pharmaceutical Benefit Advisory Committee [15]^,^ and also provide a discount of 2% (see S1 Data for analysis).

## Results

**Fig 2:**
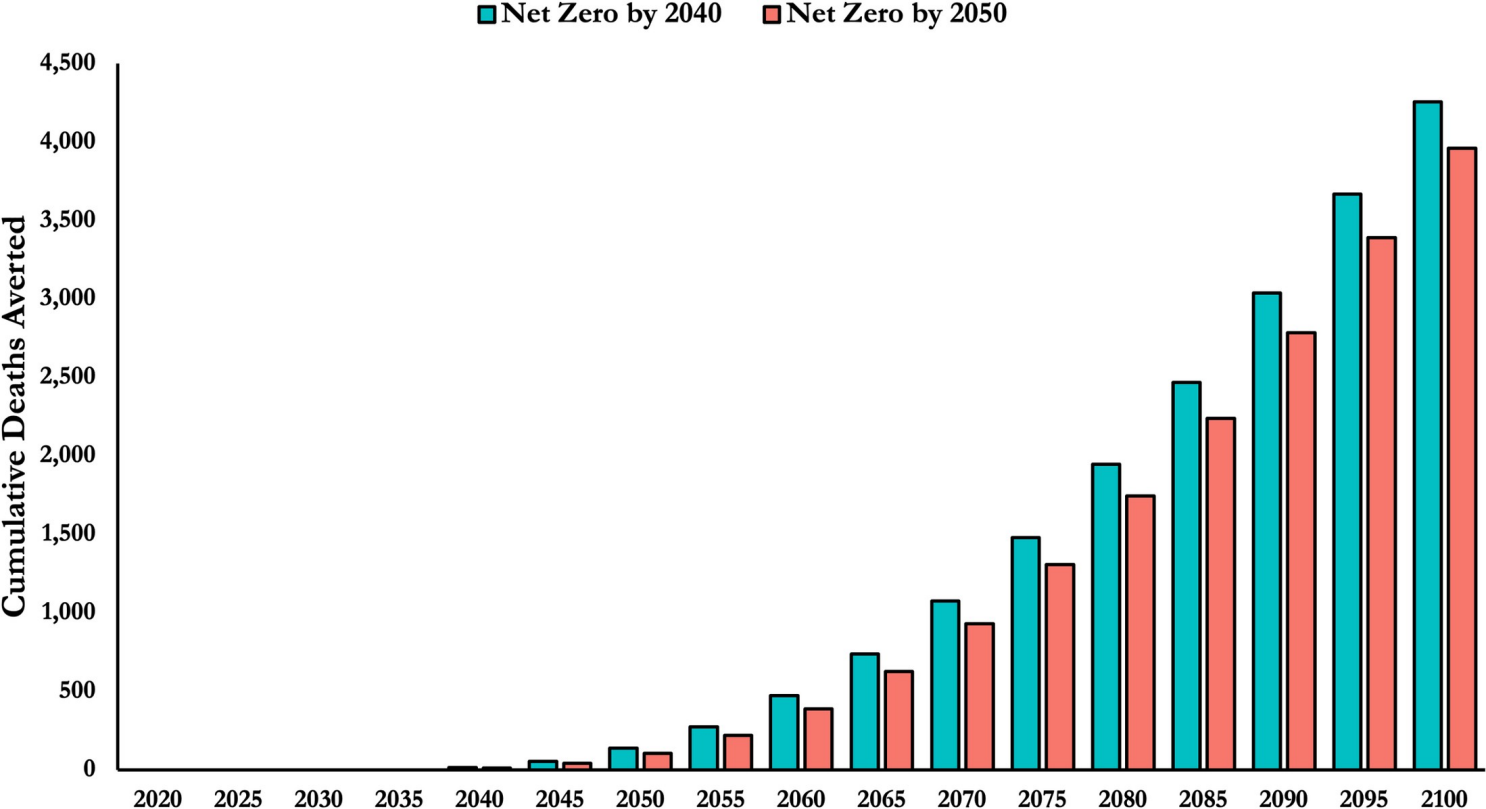
Cumulative temperature-related deaths averted from earlier decarbonisation of the health sector. The majority of lives are saved in the later part of the century.

We estimate the Australian health system will emit 4.0 Gt CO_2_-equivalent emissions less over the century compared to its BAU scenario if it achieves net zero by 2050. This translates to 69,000 fewer temperature-related global deaths from the present through to 2100 (Table 1). Most deaths are averted in the latter part of the century (as seen in Fig 2), with 74% of deaths averted between 2080 and 2100. Decarbonising by 2040 averts a total of 77,000 deaths, with 72% of deaths averted from 2080 to 2100. If the Australian economy decarbonises alongside the health sector, we project in total 988,000 fewer temperature-related global deaths with a 2050 target and 1.1 million fewer temperature-related global deaths with a 2040 target. If the global economy decarbonises by 2050, then the harm the Australian health sector causes is significantly reduced, and 8,000 deaths are averted with decarbonisation by 2040.

**Table 1:** A. The number of temperature-related deaths averted if Australia’s health system and the whole economy decarbonises by 2040 and 2050. B. The monetary equivalent welfare gain under a range of discount rates and emission trajectories.

	Australian health sector			Australian economy		
**A. Cumulative deaths averted**						
**Decarbonize by 2050** _**DICE Baseline Global Emissions Scenario**_	69,000			988,000		
**Decarbonize by 2040** _**DICE Baseline Global Emissions Scenario**_	77,000			1,101,000		
**Decarbonize by 2040** _**DICE EMRO Global Emissions Scenario**_	8,000			108,000		
**B. Monetary-equivalent welfare gain (billion $)**						
**Discount rate**	**2%**	**3.5%**	**5%**	**2%**	**3.5%**	**5%**
**Decarbonize by 2050** _**DICE Baseline Global Emissions Scenario**_	137	42.6	13.5	1955	608	192
**Decarbonize by 2040** _**DICE Baseline Global Emissions Scenario**_	151	47.1	14.9	2161	672	214
**Decarbonize by 2040** _**DICE EMRO Global Emissions Scenario**_	13.8	4.30	1.36	269	83.9	26.5

^a^DICE EMRO- DICE-Endogenous Mortality Response Optimal emissions trajectory.

We then estimate the monetary equivalent welfare gain under different assumptions for the discount rate and the global emissions trajectory. Assuming a low discount rate (2%) and the DICE Baseline global emissions scenario, the health sector averts 77, 000 deaths with a net-zero target of 2040, which represents a monetary-equivalent welfare gain of $151 billion (Table 1). At the other extreme, assuming a high discount rate (5%) and the DICE-EMR optimal emissions trajectory, the health sector averts 8,000 deaths with a net-zero target of 2040, which represents a monetary-equivalent welfare gain of $1.36 billion. If the full Australian economy decarbonises alongside the health sector, the monetary-equivalent welfare gain from the reduced mortality is $2.1 trillion with a low discount rate and the DICE Baseline global emissions trajectory.

## Discussion

We demonstrated how a decision maker may quantify the temperature-related mortality impact of embarking on various trajectories for decarbonising the health sector. A target of 2040 averts an additional 8,000 deaths compared to a target of 2050, which in turn averts an additional 69,000 deaths compared to BAU. If the Australian economy follows the health sector’s lead and decarbonises by 2040, combined they avert 1.1 million deaths compared to BAU. We converted the mortality impact into a monetary value using the VSL. If the health sector decarbonises by 2040, we estimate a gain of $151billion assuming a low discount rate and high emissions scenario. This can provide a guide for how much the health sector may be willing to spend over the next 20 years to decarbonise earlier. There is however significant uncertainty and ambiguity surrounding the actual impact GHGs will have on future health, the collective action the global economy will take to reduce their carbon intensity and debate about how we should value future health.

We can examine the annual harm to contextualise and compare GHG emissions to other pressing health priorities. Over the next decade, the annual harm attributable to the Australian health sector’s GHG emissions is near negligible, particularly when compared to the harm caused by other well established risk factors such as tobacco or high fasting plasma glucose [16]. When we look ahead however, closer to 2100, we find the harm is at par with deaths attributed to these risk factors in 2020. It is also comparable to the estimated harm that arises from medical errors [16, 17]. There are three important differences between the nature of contemporary local mortality risks and climate related mortality risks. First, the deaths predicted to occur from GHGs would primarily affect individuals in other countries, particularly Low and Lower-Middle Income countries [1]. Some have argued global harms should not be considered in climate policy analysis, such as the recent Trump Administration [18]. We disagree, recognising that climate change is a global collective action problem that requires countries to consider the full cost of their emissions, including harms on other countries when determining their own climate policy [1, 10, 11, 19]. Second, the expected harms of GHGs would primarily occur much further in the future than tobacco or sugar-sweetened beverages. Most philosophers agree that there should be no difference in the value of a life lived today and a life lived in a century, although discounting the monetized costs of premature death may be appropriate to account for individuals’ estimated rate of time preference and projected income growth [19, 20]. However some also acknowledge that discounting is permissible for uncertainty, and the ‘instrumental benefits of earlier health,’ i.e. earlier health investments can increase productivity, yielding additional value in subsequent years [21]. Economists for similar reasons look to financial markets for an estimate of a discount rate [22]. Third, the effects of climate change are much more uncertain when compared to other health risk factors [23]. We are much less confident about the impact GHGs have on mortality when compared to smoking and obesity, and also unsure about how to model and value this uncertainty, particularly tail risks [24].

Our analysis has several limitations that likely underestimate the health gains of earlier decarbonisation. DICE-EMR does not capture other mechanisms in which climate change could cause premature death outside of temperature-related mortality. For example, it does not project the mortality impact from the increased likelihood of emerging infectious diseases, flooding and civil and interstate war [10]. It also does not provide estimates for the morbidity impact of earlier decarbonisation. Reporting in Disability Adjusted Life Years (DALYs) is arguably a more useful tool for health priority setting over mortality, as it communicates a more precise and comprehensive estimate of health loss by combining years of life lost and years of life lived with the disability [21]. However, DICE-EMR is constructed based on estimating temperature-related mortality damages from the existing literature, and studies are not yet available making projections in DALYs. Additional epidemiolocal studies and projections are required to make more complete and confident mortality and morbidity estimates [25].

Others have estimated the health gains that could be realised from reduced exposure to particulate matter and other health ‘co-benefits’ of earlier decarbonisation. For example, the United Kingdom’s National Health Service (NHS) estimated decarbonisation by 2040 saves 5, 770 lives per year in reductions in air pollution and saves 38, 400 lives per year in increased levels of physical activity [26]. A more recent study derived similar estimates for the mortality co-benefits resulting from national decarbonisation by 2040 across multiple countries [27]. Health co-benefits have been incorporated in IAMs as well. For example, Scovronick et al., recently demonstrated the implications of including the health co-benefits of reduced air pollution [19]. Building on the RICE IAM, the researchers showed how mitigation nets immediate benefits globally when air pollution co-benefits are incorporated, and may make a 2 degree target consistent with what is economically optimal.

The cost of decarbonisation is also important to consider when developing policy. The health sector’s primary contributors to GHG emissions include medicines, anaesthetic gases, patient and staff transport, heating and cooling of facilities, electricity use, waste management, food and catering [28]. Hence there are multiple opportunities to reduce the health sector’s GHG production. Some opportunities are relatively simple to implement and incur no additional financial or welfare cost; for example promoting the use of low carbon inhalers or anaesthetic gases [29]. Others require significant upfront investment and planning but are forecasted to be cost saving over the long-run. For example, the NHS estimates upgrading lighting across hospitals in the UK would incur an upfront cost of £500 million but could yield a cost-saving of £3 billion over the next three decades in energy consumption [26]. Similarly, a wide range of heating, cooling and ventilation initiatives are estimated to save £250 million a year after implementation in 2034 across the secondary care estate of the NHS [26] Other costs are uncertain, such as the electrification of transport and the decarbonisation of health care product supply chains. Careful consideration of the costs and benefits of decarbonisation can help countries develop climate policy to achieve net zero emissions in the coming decades.

## Conclusion

In this paper we set out to explore the temperature-related mortality impacts of decarbonisation of the Australian healthcare system by 2040 and 2050 as compared to business as usual healthcare sector emissions. We project conservatively that the 2040 target averts an additional 8,000 deaths compared to a target of 2050, which in turn is projected to avert an additional 69,000 deaths compared to BAU. Assuming a low discount rate and high emissions scenario, decarbonisation of the healthcare sector by 2040 could provide a monetary-equivalent welfare gain of $151 billion just through the reduction in temperature-related premature mortality. Indeed, many uncertainties exist, particularly surrounding the choice of discount rate. Further, our analysis does not capture mortality gains outside of temperature-related death, morbidity gains and the co-benefits of earlier decarbonisation such as reduced particulate matter exposure and increased physical activities. Further work incorporating these impacts is likely to demonstrate greater health gains from earlier decarbonisation beyond what we have presented here.

## Supporting information

S1 Appendix (DOCX)

S1 Data Australian health sector and economy decarbonisation and temperature-related mortality damages. (XLSX)
