# Supplementary material for: The global temperature-related mortality impact of earlier decarbonization for the Australian health sector and economy: A modelling study
Source: PLoS One. 2022 Aug 3;17(8):e0271550. doi: 10.1371/journal.pone.0271550 (PMC9348697; doi:10.1371/journal.pone.0271550)
Supplement: S1 Appendix — (DOCX) [file pone.0271550.s001.docx]

# S1: Appendix 1

## **DICE 2016: Projected GHG emissions and climate sensitivity**

The DICE-2016 model integrates a macroeconomic model with a climate model to simulate the interplay between the climate and the economy.^1^ It forecasts cumulative GHG emissions ($E$_GC_), as depicted by the following equation:

$E$_GC_= $\sum_{2100}^{2020} \sigma(\tau)[1-\mu(\tau)]\Upsilon(\tau) + E$_Land_ $(\tau)$

$\Upsilon(\tau)$is gross economic output each year, representing a Cobb-Douglas function of capital, labour and technology. GDP per capita is projected to increase at 2.1% per year from 2015 to 2050, and 1.9% from 2050 to 2100.  $\sigma(\tau)$represents the level of carbon intensity of economic output each year, which decreases by $\mu(\tau)$, the emissions reduction rate. The DICE Baseline global emissions scenario assumes the rate of decarbonization is -1.5% per year. CO2 emission projections from land-use ($E$_Land_ $(\tau)$) are based on results from the Fifth Assessment of the IPCC and other modelling groups, contributing about 3Gt of CO2 a year.

DICE 2016 has a three-reservoir climate model to capture the carbon cycle, and the subsequent feedback on surface temperature. The parameters for the equations are informed by recent Earth system models using a Bayesian approach - doubling of CO2 generates a mean warming of 3.1 ℃, aligning closely with the Fifth Assessment of the IPCC.

## **DICE EMR: Systematic research synthesis of the climate mortality literature**

As described in the Nature Communications study, *The Mortality Cost of Carbon*, The DICE-EMR damage function is estimated from projections made in the scientific literature that were chosen through a systematic research synthesis. Google Scholar was used to identify studies that estimated the mortality effects of climate change with the following search terms: climate change AND mortality AND Death AND Global AND Projection.^2^ Google Scholar was considered the most appropriate search engine given climate studies were interdisciplinary in nature, and Google Scholar best scans literature from multiple disciplinary databases. The search was conducted in September 2019 and Google Scholar was programmed to only consider studies published in the last twenty years. The first 100 studies were examined after sorting the search results by relevance. Certain criteria were used to find studies of a high quality. They included:

1. Provides a projection of the number of excess deaths or the increase in the mortality rate for a specific warming scenario or scenarios.
2. As comprehensive as possible of the human mortality impacts caused by climate change.
3. Mortality estimates are projected net of defensive adaptation.
4. Mortality estimates are aggregated at the global level, or global estimates can be derived from the provided estimates.
5. Published in the last 20 years.

After cataloguing 100 studies (see the supplementary materials of the *Mortality Cost of Carbon* for full details and replication), only three studies were deemed to have sufficiently met the criteria to be included in constructing a mortality damage function: a 2014 WHO Report Quantitative risk assessment of the effects of climate change on selected causes of death, 2030s and 2050s,^3^ a 2019 Climate Impact Lab (a collaboration between the University of Chicago, University of California Berkeley, and Rutgers) report Valuing the Global Mortality Consequences of Climate Change Accounting for Adaptation Costs and Benefits,^4^ and a Lancet Planetary Health article 2017 Projections of temperature-related excess mortality under climate change scenarios.^5^

The 2014 WHO report projects global excess mortality from a wide variety of channels including undernutrition, malaria, dengue, diarrheal disease, and heat in 2030 and 2050. It accounts for adaptation in the mortality projection from heat, although the effects of adaptation in the undernutrition and disease-related risks appear to be limited. The authors emphasize that despite their efforts to quantify important mortality pathways, their estimates of the future mortality effects of climate change remain incomplete because they could not calculate other pathways including river flooding, water scarcity, and conflict.

The 2019 Climate Impact Lab Report uses an econometric strategy that exploits historical variations in temperatures to find a relationship between mortality and temperature in regions across the globe. They exploit spatial heterogeneities in the mortality-temperature relationship to understand the role that different income levels and demographics play in affecting the climate-mortality relationship. They break the world into 24,378 regions and project incomes, populations, and climate into the future to estimate excess mortality that results from climate change in these regions. Importantly, their approach allows them to account for the benefits of higher incomes and climate adaptations to gain a more accurate estimate of the effect of climate change on mortality accounting for future adaptation. Bressler utilised their reduced-form global projection of the effect of climate change on mortality accounting for adaptation. The Climate Impact Lab’s approach allows them to account for climate-mortality effects that are driven by direct changes in the short-run distribution of temperatures such as the net mortality effect of more hot days and fewer cold days, the mortality effect of increased surface ozone formation, and even the effect of hot days on murders and suicides. However, their approach arguably does not fully capture climate-mortality channels that are driven in part by longer-term pathways that are not econometrically identified from shorter-term temperature fluctuations such as some diseases, flooding, and undernutrition.

The 2017 Lancet Planetary Health Report uses a dataset of daily observed mean temperature and mortality counts from locations around the globe from 1984-2015 to estimate temperature-mortality relationships. They project excess mortality for cold and heat and their net change in a number of locations around the globe under RCP 2.6, 4.5, 6.0, and 8.5. Given their statistical strategy, this report has similar limitations to the 2019 Climate impact lab report: climate-mortality effects driven by direct changes in the distribution of temperatures are likely to be captured, but more complex climate-mortality channels such as changes in contagious diseases, flooding, and the effect on food supply are unlikely to be captured. Among the three studies, this study was the most borderline as to whether it would be included in Bressler’s model. While the study does include projections for a number of locations around the globe, it does not cover all of the world’s population. It covers 9 regions that include all the Americas, Europe, Australia, East Asia, and South East Asia. This represents about 40% of the world’s projected population in 2050. Importantly, the study is missing data for regions that are expected to bear the most severe climate change mortality impacts: South Asia, the Middle East, and Africa. To project a global mortality estimate from this report, Bressler used the 2019 UN population prospects projections for the percentage of the world population that is expected to reside in each of the 9 regions used in the report in 2055 and 2095. Bressler then calculated the world population residing in each of the 9 regions as a percentage of the total projected population in each of the 9 regions in 2055 and 2095 so that this percentage for each of the 9 regions adds to 100%. Bressler then multiplied this percentage by the expected percentage increase in the mortality rate in the region given in the report to create a population-weighted global estimate of the increase in the mortality rate. However, this is an underestimate of the global mortality impact because the original paper leaves out projections for South Asia, the Middle East, and Africa. In addition, unlike the 2019 Climate Impact lab report, this report does not assume adaptation changes. Although this violates one of our idealized criteria, economics literature on climate-mortality adaptation has suggested that in the United States, there has already been significant adaptation to climate change that has ameliorated the mortality effect of hot days, in particular through the adoption of air conditioning. This has likely already occurred in other rich regions that have widely adopted air conditioning, such as in Europe, much of the Americas, and some countries in East Asia. Much of the expected future benefit of climate-mortality adaptations can be expected to come from emerging countries that adopt air conditioning. The exclusion of the most vulnerable regions contributes towards understating the future global mortality projection while the exclusion of adaptation contributes towards overstating the future global mortality projection. Utilizing the methodology described above, the 2017 Lancet Planetary Health report projects that in RCP 8.5 in 2100, climate change causes a 4.0% increase in the mortality rate. The 2019 Climate Impact Lab Report makes a global projection and accounts for adaptation, and they project that in RCP 8.5 in 2100, climate change causes a 6.6% increase in the mortality rate. Given similarities in the methods of the two reports, this suggests that the net effect of excluding the most vulnerable regions and excluding adaptation may be to understate the risk of mortality.

Bressler also performed sensitivity analyses around the inclusion of the three studies to estimate the mortality damage function. He conducted one mortality damage function specification that excluded estimates from the WHO study and another mortality damage function specification that excluded estimates from the Lancet Planetary Health study. In both cases, he found that this increased mortality cost of carbon and social cost of carbon estimates.

## **Curve Fitting the mortality damage function**

A scatterplot of the central estimate increase in the mortality rate as a function of the increase in global average temperatures from the studies used is shown below.

**Supplementary Graph 1: Green dots are estimates from Gasparrini et al 2017, purple dots are estimates from Hales et al 2014, blue dots are estimates from Carleton et al 2019. Produced in Stata.**

Bressler estimated the mortality damage function by fitting a curve through this data. This was done by running a weighted regression where each study is given 1/3 weight, and each data point within a study is given proportional weight. Bressler ran this weighted regression using Matlab’s curve fitting tool for a number of functional forms, shown in supplementary figure 3 and supplementary table 3. These include (A) linear, (B) quadratic, (C) 3^rd^ order polynomial, (D) exponential, (E) power, and (F) two-parameter Weibull. As supplementary figure 3 and supplementary table 3 show, the linear curve (A) produces a relatively poor fit while each of the non-linear functional forms (B-F) produce similar curves that provide an excellent fit. To maintain consistency with the functional form of the climate-economy damage function in the original DICE model, Bressler chose to use the quadratic functional form.

**Supplementary Table 1:** **Mortality Damage Functional Form Sensitivities.** Each of the non-linear functional forms provide a strong and similar fit to the central estimate data

1

**Supplementary Figure 1: Mortality Damage Functional Form Sensitivities.**
